# Leveraging the Patient and Family Voice in the Development of Patient Education: Supporting the Pediatric Oncology Experience

**DOI:** 10.3390/cancers17071201

**Published:** 2025-04-01

**Authors:** Anna M. Jones, Alyssa Marchetta, Kendra R. Parris, R. Elyse Heidelberg, Niki Jurbergs

**Affiliations:** St. Jude Children’s Research Hospital, Memphis, TN 38105, USA; alyssa.marchetta@stjude.org (A.M.); kendra.parris@stjude.org (K.R.P.); elyse.heidelberg@stjude.org (R.E.H.); niki.jurbergs@stjude.org (N.J.)

**Keywords:** childhood cancer, psychosocial care, psychoeducation, patient education, patient-family collaboration, anticipatory guidance

## Abstract

Given the understandable patient and caregiver distress that often fluctuates across treatment, especially at diagnosis and with major disease milestones, it is important to prioritize interventions that are known to best support families during these key times of transition. Individualized and time-sensitive psychoeducation and anticipatory guidance are effective interventions in reducing distress and fostering adaptive coping across the cancer trajectory and are a standard for the psychosocial care of children with cancer and their families. Patient education materials have proven to be a flexible, accessible, cost-effective, and easily adaptable tool for delivering these interventions to this population. Moreover, within the field, there has been a call to action for the inclusion of patients and families in the development of research study design and clinical programming, recognizing them as the experts of their own experiences. This commentary aims to explore how patients and families can be actively involved in the creation, assessment, and implementation of patient education materials, fostering a collaborative partnership between families and clinicians. We provide detailed examples of this collaboration in the creation of patient education materials focused on promoting coping at diagnosis, supporting siblings and assisting with social reintegration at the end of treatment, promoting responsive caregiving for young children, and supporting caregiver coping through the creation of a podcast. Ultimately, this commentary seeks to emphasize the importance of partnering with patients and families to ensure their voices remain central to intervention development and implementation.

## Simple Summary

The inclusion of patient families in clinical program development and research design has received growing recognition in recent years. While the existing literature highlights broad involvement methods, such as focus groups and patient and family advisory councils, detailed examples of how these collaborations shape intervention development and execution—particularly in the context of patient education materials in pediatric oncology—remain limited. This commentary aims to highlight the importance of leveraging the patient and family voice in the development of patient education materials to further support the pediatric oncology experience. By examining the research to date and offering concrete examples of these collaborations, the authors hope to highlight not only practical approaches but also the critical importance of keeping the patient and family voice at the center of this work.

## 1. Background

Approximately 15,000 children and adolescents in the United States are diagnosed with cancer each year [1]. Understandably, the receipt of a childhood cancer diagnosis is often met with heightened distress by the child and their family [2]. With appropriate psychoeducation and anticipatory guidance, as well as time to process the diagnosis and determine a treatment plan, distress levels decrease [3,4,5,6]. This is not to say that heightened distress is only present at diagnosis. It is not uncommon for levels of distress to ebb and flow throughout treatment, with major milestones and transitions [7,8,9,10]. In fact, the Standards for the Psychosocial Care of Children with Cancer and their Families [11] tout the importance of access to anticipatory guidance and psychoeducation to promote adaptive coping across the cancer care continuum [12].

The Pediatric Psychosocial Preventative Health Model (PPPHM) [13] provides a framework for understanding and stratifying psychosocial risk using three distinct tiers, which then guides intervention implementation. Those at the lowest risk are captured within the universal level (i.e., exhibiting expected levels of distress); those at moderate or high risk fall within the targeted (i.e., elevated distress in the context of identified risk factors) or clinical level (i.e., significant and persistent distress), respectively. This screening approach allows for tailored interventions based on psychosocial risk, such that targeted (e.g., collaborative identification of adherence barriers and strategies to improve adherence) or clinical (e.g., cognitive-behavioral therapy for anxiety) interventions can be offered alongside universal interventions when indicated [13]. Recent findings suggest that the implementation of this universal screening process followed by personalized care, such as psychoeducation at the universal level, is crucial in promoting equitable access in pediatric health care [14]. Further, systematically delivering psychoeducation serves as a platform for meeting the related standard for psychosocial care of patients and families facing pediatric cancer [12].

### 1.1. Benefits of Using Patient Education to Facilitate Psychoeducation

One tool for supporting the delivery of psychoeducation is patient education materials. This mechanism for intervention implementation offers many benefits, including portability, affordability, broad reach, and tailorability.

#### 1.1.1. Portability and Flexibility of Format

Patient education materials can be shared in a paper-based format (e.g., booklet, handout) or electronically (e.g., through the EHR, e-mail, website, QR code). The ability to offer these materials in various formats increases their accessibility and portability [15], allowing for a greater number of patient families to obtain and benefit from these interventions.

#### 1.1.2. Affordability and Cost-Effectiveness

Additionally, compared to other intervention modalities, patient education materials are relatively low in cost and resource burden [16]. There is also a plethora of data indicating a positive return on investment of patient education materials, suggesting a cost-effective and impactful avenue for universal intervention [17].

#### 1.1.3. Reach

Efforts to more consistently use a plain language editing process have improved comprehension and uptake of information and recommendations, increasing access to those with limited health literacy [18]. Further, with advances in technology, there is now the ability to translate web-based patient education materials into additional languages [19].

#### 1.1.4. Tailorability

When considering childhood development broadly, there is uniform recognition of unique differences and needs of children across developmental stages as they make their way toward adulthood [20]. It is important for this to also be reflected in patient education materials. Content can, and often should, be adapted to address the unique needs of patients across developmental stages (e.g., infancy, early childhood, school-age, adolescence, etc.), ensuring applicability and appropriateness of anticipatory guidance and psychoeducation provided through these materials [12].

### 1.2. The Patient and Family Voice

There has been a relatively recent recognition of the importance of the inclusion of patient families in the development of clinical programming, as well as research study design [21]. This has led to a wider representation of patient families within national and international scientific and clinical societies. Further, as patient families are the experts on their experiences, there has been an uptick in the creation of patient and family advisory councils and boards to review and provide comment on institutional and clinical initiatives [22]. The engagement of patient families as key collaborators in the development of patient education materials is paramount in facilitating the acceptability of these interventions and optimizing their benefits. While there is a dearth of literature outlining established frameworks for patient-family engagement in the development of patient education materials specifically, the extant literature provides frameworks that can be adapted for this purpose. The Patient-Centered Outcomes Research Institute (PCORI) details six core expectations for patient-family collaboration in research. These include: (1) broad representation, (2) continuous engagement of patient-family collaborators at each step in the research process, (3) funding for patient-family engagement in research, (4) leveraging unique skillsets and providing training/support for teamwork, (5) the inclusion of patient families in decision making, and (6) eliciting feedback assessing engagement [23]. While developed specifically for patient-family engagement in research, many of the core principles within PCORI can be adapted to patient-family engagement in clinical programming. Considering intervention development more specifically, intervention mapping is a process that was developed to engage patient families and key collaborators in intervention and program development. It is comprised of six unique steps: (1) conducting a needs assessment, (2) identification of objectives, (3) theoretical framework/justification for the chosen intervention, (4) intervention design and planning, (5) intervention implementation, and (6) intervention evaluation [24]. These steps can be applied to the development of various interventions, including the creation of patient education materials to deliver psychoeducation. Although this commentary does not represent original research or quality improvement methodology, it seeks to outline ways in which patient families can be actively engaged in the development, evaluation, and implementation of patient education materials through an intervention mapping approach, representing a partnership between patient families and clinical staff.

## 2. Collaborative Needs Assessment and Identification of Objectives

Prior to the development of any patient education materials, it is strongly recommended that an assessment with key collaborators be conducted to ensure the materials developed will meet the needs of and be acceptable to the individuals who will be providing, as well as receiving, the intervention [25]. Clinicians are likely to be knowledgeable about various content domains and are responsible for considering theoretical frameworks for intervention development, as well as the theoretical and empirical justification for the chosen intervention. Collaboration between clinicians and the patient’s family is critical in identifying which aspects of a content area are likely to be most needed, desired, and impactful to patient families [21]. For example, while *all* aspects of sleep hygiene are important, patient families may identify that there is one aspect that is universally challenging that they would like to prioritize (e.g., balancing a consistent sleep/wake schedule in the context of medications that frequently induce drowsiness). The collaborative needs assessment can take many forms (e.g., patient-family advisory council, focus group, online advisory community), and the modality for the assessment largely depends on which avenues are accessible to the clinician. The needs assessment should be conducted once an avenue is selected for patient-family engagement and prior to intervention development [24]. Importantly, following the needs assessment, ongoing collaboration with patient families at each step of intervention development, implementation, and refinement is essential in creating interventions that are seen as acceptable to patient families [25].

In addition to the collaborative identification of the most salient content areas for patient education materials, adequate theoretical justification for intervention development is paramount to the effectiveness and utility of the intervention [24]. Further, ongoing conversations with collaborators, especially patient-family collaborators, are important in guiding other aspects of intervention development. These include when the intervention should be delivered, how the intervention should be delivered, and the target audience for the intervention. The timing of intervention delivery within the cancer care continuum is critical to the uptake of patient education materials and the optimization of intervention effectiveness [26]. For example, the consideration of a patient and family’s cognitive load is imperative in intervention timing [27]. As such, collaboration among psychosocial disciplines to minimize duplication of services and protect against the redundancy effect, as well as to spare cognitive load, is critical. This allows patient education interventions provided by each discipline to be optimally consumed and utilized. Additionally, the timing of the intervention should be informed by when the information is likely to be most beneficial for patients’ families. For example, patient education outlining strategies for coping with radiation therapy should ideally be given prior to the start of radiation therapy but not so far in advance that families may not recall the information provided.

Furthermore, content area, target audience, and patient-family collaborator input should be considered when determining what amount of information should be included and how it should be presented [16]. If the content is being created for consumption by children, emphasis on graphics over written text is likely to be most appropriate and beneficial [28]. Additionally, there has been some literature to suggest increased involvement in intervention consumption by children when there is a gamified component to the intervention [29].

## 3. Collaborator Engagement in Intervention Development, Implementation, and Evaluation

The partnership between clinicians and patient families should extend beyond the initial collaborative needs assessment. The inclusion of patient families on the intervention development team is crucial to creating an intervention that will be acceptable to and meet the identified needs of patient families while being grounded in evidence-based practice [21]. Although the engagement of one patient or family member in informing intervention development may lead to novel insights not thought of by others on the development team, the absence of input from multiple patient families is likely to decrease the generalizability of the intervention to a broad range of patient families [30,31,32]. For example, the inclusion of a caregiver of a school-aged child on the development team would provide excellent insight based on this patient family’s experience; however, it would not be representative of caregivers of all school-aged children and certainly not representative of caregivers of young children or adolescents. As such, the engagement of diverse patient families in intervention development is essential in optimizing the acceptability and appropriateness of the intervention for a broader audience. Diversity in representation should be considered broadly and may include age, sex, gender, primary language, cancer diagnosis, and socioeconomic status, among others. The avenue for engaging patient families in intervention development, implementation, and refinement processes will depend on which modes are available to the clinician. Below, three potential avenues are discussed, including the benefits and drawbacks of each approach (see Table 1 for direct comparisons). As emphasized previously, patient families should be involved from the beginning of the intervention development process, starting with the needs assessment, with continued involvement through intervention evaluation and optimization [24].

One mechanism for gathering input from a wide variety of patient families is through focus groups [31]. Focus groups allow for discussion among multiple patient families. These discussions can be guided, with set questions posed by the moderator of the focus group, or open, with little input and direction from the moderator [33]. Focus groups offer the opportunity for diverse patient families to discuss and offer input and direction on intervention development. As multiple patient families are present, focus groups offer the advantage of an idea or thought to be triggered by something another patient family says, therefore, enriching the discussion and taking the conversation in directions it may not have gone otherwise. While there are many positive aspects to focus groups, drawbacks can also be present. Focus groups are frequently resource and time-intensive, both in terms of scheduling and facilitating, as well as in synthesizing the information shared [34]. Further, depending on the dynamic of the group, some patient families may feel uncomfortable sharing their perspective, which would then potentially limit the generalizability of the information gleaned during the group [35].

Another system for receiving ongoing input from patient families is through patient-family advisory councils (PFACs). PFACs typically support the mission of hospitals by providing a family voice in health care decision-making to improve quality, safety, and experience of care [36]. PFACs promote an environment where families and professionals work together to ensure optimal patient care, and they represent an effective mechanism to partner with families to develop and implement new programs and interventions. Frequently, there are efforts to ensure PFACs are representative of the patient population and include patient families from various backgrounds [37]. PFACs can be time intensive and require significant commitment from patient families, which may preclude the involvement of some families.

Lastly, online patient-family advisory communities offer a unique approach to soliciting feedback from a large group of patient families. These can be constructed in a multitude of ways. Online advisory communities can be structured to allow for free-flowing conversation among members of the community, often overseen by a moderator of the forum [38]. The moderator can pose specific questions or elicit feedback on a particular topic. Members of the online advisory community may also be able to offer feedback or input unprompted, should the forum be designed in this way. Other online advisory communities are structured with patient families as respondents to more traditional surveys [39]. While this method allows patient families to respond to calls for feedback and input at their leisure and frequently with the option for anonymity, one significant drawback is the inability to engage in back-and-forth discussion among the group of patient families or between patient families and the clinician or researcher eliciting feedback. Further, should the input be provided anonymously by a member of the online advisory community, this precludes clinicians and researchers from seeking clarification or asking follow-up questions of specific individuals within the online advisory community.

**Figure 1:**
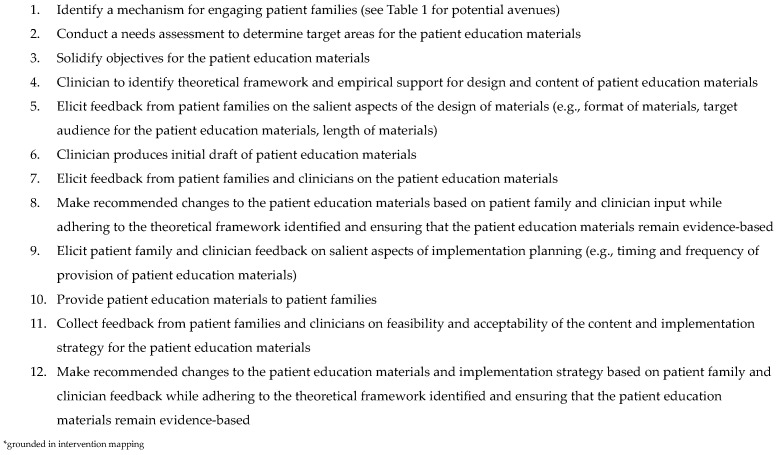
Steps for Collaborative Development of Patient Education Materials *.

As no one avenue for patient-family engagement is likely to provide a comprehensive picture of the patient-family voice, utilization of multiple methods for patient-family collaborator engagement and input is recommended [25]. In addition to patient-family involvement in crafting the intervention, it is important to elicit feedback and input again from patient families once the intervention development is complete and prior to its implementation. Further, ongoing feedback following intervention implementation using the above-mentioned avenues for engagement to determine acceptability and incorporate additional feedback, in turn, optimizing the intervention, is paramount to its success [23]. See Figure 1 for a list of steps to consider in collaborating with patient families in the development of patient education materials.

## 4. Putting It into Practice: Examples of Patient-Family Collaboration in the Creation of Patient Education Materials

At our institution, nine steering councils (or subcommittees) report to the PFAC, with the staff champion and caregiver lead of each council sitting on the PFAC. This ensures an optimal bi-directional flow of communication between the PFAC and the steering councils. The focus of the steering councils spans hospital efforts, with some examples including patient experience, caregiver support, quality and patient safety, and nursing. The Patient Education Steering Council is comprised of eight caregiver advisors and three staff members who are intimately involved in hospital communication and education efforts, such as development of a learning management system for caregivers, creation of educational content for an online resource platform, review of written educational materials, and development of educational videos for caregivers about infection control, medication safety, and other treatment-related topics. The engagement of caregiver advisors through the Patient Education Steering Council and other councils has contributed directly to hospital planning, evaluation, and policy to improve the care of patients and to guide best practices. Our institution is also fortunate to have a diverse and active online advisory community. We have partnered with patient-family collaborators on the PFAC, through online advisory communities, and as formal project team members in the creation of patient education materials to promote coping throughout the illness trajectory (i.e., at diagnosis and completion of cancer-directed therapy) and to support the unique needs of patients and their caregivers.

The examples of patient education materials developed at our institution are discussed below and are representative of a universal intervention for patient families within the PPPHM. While the patient education materials described next are offered to all families when meeting with psychology at specified timepoints in the cancer care continuum (i.e., at diagnosis and end of therapy, at admission to the hospital, as indicated), they are also readily available in our waiting room to any interested patient family and digitally to patient families outside of our institution through together.stjude.org. These materials are available in printed form in English and Spanish and digitally in 12 languages. Although patient-family feedback has yet to be collected in a systematic fashion regarding these materials, initial anecdotal feedback has been uniformly positive. Please see Supplemental Material for hyperlinks to these published patient education materials.

### 4.1. Patient Education Materials to Promote Coping and Adjustment at Diagnosis

Patient education materials outlining strategies to support psychosocial health during treatment were developed as a component of our clinical program that offers universal psychological assessment and intervention to all oncology and transplant patients near the time of diagnosis. The patient-family voice was central to the development of this program, with aspects of intervention mapping utilized (i.e., interventions grounded in theory and best practice, patient-family engagement in program development and evaluation). The PFAC provided support for the program, as well as guidance regarding the scope of services offered within the program. Building upon advisement from the PFAC, patient education materials were created with input from subject matter experts, including pediatric psychologists who specialize in assessment and treatment across developmental stages (i.e., early childhood, school age, adolescence and young adulthood) and clinical domains (e.g., sleep, pain, etc.) in pediatric oncology. In addition to clinical training and practice, content derived from subject matter experts was born out of years of input received from patients’ families who were willing to share the challenging aspects of their oncology journey. These patient education materials were developmentally tailored and created to augment in-person intervention, with the goal of sharing broadly applicable strategies to promote patient coping and adjustment near the time of diagnosis and throughout cancer-directed therapy. Following the launch of this clinical program and the implementation of the patient education materials, we leveraged our online advisory community to examine the patient-family perspective of psychology services near the time of diagnosis. Please see Bernstein et al. [40] for a comprehensive description of methods and results. Caregivers identified multiple psychosocial areas to be very important or somewhat important [40]. While many of these domains are addressed in current versions of our patient education materials, the patient-family perspective will shape the development of future education to support patient families at diagnosis.

### 4.2. Patient Education Materials to Support Sibling Coping at End of Treatment

Consistent with the recognized need for systematic psychosocial support at various points throughout the cancer care continuum, a new institutional program aimed at providing support to patient families during early survivorship was created. Shortly following the launch of this program, a survey was sent out to the patient-family online advisory community seeking feedback on the quality of support received during the transition off therapy. Forty-three caregivers completed the survey. One tangible need identified through this survey that was still unmet with the creation of the early survivorship program was systematic support for siblings during this transition. This need was echoed by 63 caregiver respondents in another survey sent to the patient-family online advisory community seeking insight on the patient-family experience with psychological and psychosocial support during the transition off therapy. As such, patient education materials were created by a team of psychosocial providers (i.e., psychologists, social workers, child life specialists) to address this identified gap. While general strategies were included in these patient education materials to support siblings of all ages, additional developmentally tailored strategies were also provided for young children and adolescents. This educational intervention is provided to all families of patients with siblings when meeting with psychology prior to the transition off therapy. Consistent with aspects of an intervention mapping approach, ongoing efforts to engage patient families in intervention evaluation and optimization are needed.

### 4.3. Patient Education Materials to Support Social Reintegration at End of Treatment

A survey was sent to the patient-family online advisory community to seek feedback and inform the optimization of existing services aimed at supporting patient social reintegration following completion of cancer-directed therapy and/or return to the child’s home community. This survey sought to better understand the positive and negative aspects of current processes and mechanisms for supporting social reintegration, as well as allow families to provide suggestions on how our institution might improve these services. Results from caregivers (N = 55) provided meaningful guidance on the development of patient education materials outlining strategies to promote positive social development and functioning during this reintegration. This education was developmentally tailored and shared with all patient families when meeting with a psychologist at the time of transition off therapy and/or back into the home community. Consistent with aspects of intervention mapping, plans are in place to evaluate this intervention’s acceptability and seek additional patient-family collaborator feedback to optimize the effectiveness of the patient education materials.

### 4.4. Patient Education Materials to Promote Responsive Caregiving for Young Children

Guided by the literature and an identified need, an institutional quality improvement project was initiated to increase responsive caregiving for infants and toddlers during hospitalizations [41,42]. The patient-family voice was essential at every step of the process, and an intervention mapping approach was utilized. A caregiver from the PFAC served as a member of the core project team, offering insight from her experiences. Prior to the development of any interventions to promote responsive caregiving, the project team presented to the PFAC for general guidance and recommendations. Additional information regarding caregiver experiences during their young child’s hospitalizations was gleaned from caregiver responses (N = 18) to a survey distributed through the patient-family online advisory community. Feedback indicated that a substantial portion of caregivers did not receive education on the importance of responsive care or safe provision of responsive care (e.g., how to safely hold, diaper, or feed) for their child, but such education would have been helpful. As such, several educational interventions were collaboratively developed and reviewed by subject matter experts (e.g., nurses, critical care physicians, child life specialists, and psychologists) and caregivers. Caregiver input also guided the implementation of these interventions, which are provided to all families of patients under age 3 years at the time of inpatient hospital admission via electronic healthcare record and via display on the TV screen in the patient’s room. The very positive impact of these interventions in increasing responsive caregiving behaviors was measured using a QI framework, and findings are under preparation for hopeful publication. At the suggestion of the caregivers involved in this project, the next educational intervention being developed is aimed at training bedside nurses to provide individualized in vivo education and modeling to families during hospitalization.

### 4.5. Collaborative Creation of a Podcast to Support Caregiver Coping

As part of ongoing efforts to enhance caregiver support programs and consistent with an intervention mapping approach, the institutional caregiver support committee surveyed our patient-family online advisory community. Questions were posed regarding the acceptability, feasibility, and utility of various caregiver support programs being considered for development. One hundred fifty-nine caregivers completed the survey. While most respondents indicated interest in support groups and social events, they also reported attendance barriers, including timing of the event, illness of their child, and lack of childcare. Moreover, many caregivers indicated that informational and educational support available through a podcast would be desirable and would eliminate most of these barriers. As such, the committee partnered with key collaborators in hospital administration and medical communications to begin the development of a podcast series for caregivers of children with serious illnesses. Patient-family collaborators were directly involved in content development, the selection of staff moderators and caregiver participants, and the naming of the podcast. Caregivers S.H.A.R.E. aims to support, honor, advise, reflect, and encourage. The content presented in the first two seasons included navigating a new diagnosis, handling logistics during treatment, seeking support, maintaining healthy relationships during treatment, supporting siblings, parenting during illness, and transitioning off active medical treatment. Consistent with both the mission of the institution and the universal level of the PPPHM, the podcasts are distributed widely on most major podcast platforms and are available to caregivers around the world. To date, the podcast has been downloaded 1746 times and in 21 countries.

## 5. Conclusions

The extant literature suggests that patient education materials are an effective means of providing universal interventions, including anticipatory guidance and psychoeducation, for pediatric oncology patient families [12,16,43]. There are multiple benefits to using patient education materials to provide psychoeducation to patient families, including the portability, affordability, reach, and tailorability of these interventions [12,15,16,18]. Additionally, the use of patient education materials to provide psychoeducation is consistent with the PPPHM and the Standards for the Psychosocial Care of Children with Cancer and their Families [11,13]. While patient education materials offer a robust mechanism for intervention delivery, the success of these interventions depends on their acceptability by patients’ families [15,16,18,43]. As such, it is critical to involve patient families in the intervention development process from the beginning, including in choosing content, working through logistical considerations, and intervention development and implementation [24]. Engagement with patient-family collaborators can take many forms, such as through engagement with PFACs, conducting focus groups, seeking input from online advisory committees, and having patient families as formal members of the project team. Intervention mapping is a recommended empirical approach to collaborative intervention development with patient families that should be considered [24].

While the benefits of using patient education materials in the provision of psychoeducation are significant [12,16,43], limitations must also be acknowledged. Patient education materials require routine review and revision to ensure that materials are in line with current evidence and best practices. Further, it can be challenging to create patient education materials that are broadly applicable to a wide audience while also acknowledging unique cultural factors and lived experiences of individuals. This highlights the importance of having diverse representation from patient-family collaborators in the intervention development process. Further, there are often individualized preferences for the delivery of patient education materials, with some individuals expressing a preference for tangible written education, while others desire to receive this information electronically or audio-visually [43]. Many of these limitations can be minimized by clinicians coming alongside patient families when providing psychoeducation through patient education materials to flexibly adapt and individually tailor the psychoeducation to meet the needs of each patient family.

An important limitation to note is that this commentary does not represent original research or quality improvement methodology but seeks to encourage engagement with patient families in the creation of patient education materials, with recommended steps provided based on the extant literature [24] and tangible examples of how this has been accomplished at our institution. Strict adherence to intervention mapping was not utilized in the development of patient education materials at our institution, and we recognize that this is a limitation in drawing specific and robust conclusions from the examples provided. As such, the conclusions presented are based on the extant literature. Ongoing assessment of patient education materials at our institution presented as examples in this commentary is needed to empirically examine the acceptability and effectiveness of these interventions. Further, examples provided are based on the experience of one institution, which may limit generalizability. For those interested in engaging families in the development of patient education materials, Figure 1 provides a recommended process grounded in intervention mapping.

Going forward, continued collaboration with patient families to guide the expansion and refinement of established patient education materials is essential. Further, while there has been increased attention to the incorporation of the patient-family voice in clinical efforts, there are still areas for improvement. Formalized guidelines for the incorporation of patient families in *all* aspects of clinical programming and intervention development are essential to ensure that patient families have a voice in aspects of their care that will directly impact them and are the next step in systematically including patient families in this way.

## Figures and Tables

**Table 1:** Avenues for Patient-Family Engagement.

Avenue	Benefits	Drawbacks
Focus Groups	Discussion among multiple patient families Diverse family perspectives Ideas/thoughts may be triggered during the group based on something another participant shares Discussions may be guided or open-ended	Resource intensive Time intensive Potential participant discomfort sharing opinions in a large group
Patient-Family Advisory Councils (PFACs)	Institutionally supported Aim to be representative of the patient population Diverse family perspectives Discussion among multiple patient families	May not always be representative of the patient population Time intensive Resource intensive
Online Advisory Communities—Forum	Specific questions able to be posed Allows for unsolicited feedback Patient families can provide feedback at their leisure Wider reach Possibly more representative of the patient population Not time intensive for patient families	Resource intensive (moderator) Time intensive (moderator) Difficult to regulate and for moderator to respond to potentially harmful comments in real time
Online Advisory Communities—Surveys	Patient families can provide feedback at their leisure Allows for anonymity Responses are structured and answer specific questions Allows for a variety of methods for obtaining feedback (e.g., likert scale, ranking, open-ended) Wider reach Possibly more representative of the patient population	Clinicians cannot follow up on anonymous replies for clarification No back-and-forth discussion among participants

## Supplementary Materials

The following supporting information can be downloaded at: https://www.mdpi.com/article/10.3390/cancers17071201/s1, Published patient education materials.
