# Supplementary material for: Leveraging the Patient and Family Voice in the Development of Patient Education: Supporting the Pediatric Oncology Experience
Source: Cancers (Basel). 2025 Apr 1;17(7):1201. doi: 10.3390/cancers17071201 (PMC11988127; doi:10.3390/cancers17071201)
Supplement: Supplementary file 1 [file cancers-17-01201-s001.zip › cancers-3518051-supplementary.pdf]

Supplemental material

Patient education materials to promote coping and adjustment at diagnosis

Young Children

https://together.stjude.org/en-us/emotional-support-daily-life/parents/how-to-support-your-young-child-during-illness.html

School-Age Children

https://together.stjude.org/en-us/emotional-support-daily-life/parents/how-to-support-your-school-aged-child-during-illness.html

Adolescents & Young Adults

https://together.stjude.org/en-us/teensand20s/take-care-of-yourself/coping-with-serious-illness-tips-for-teens.html

Patient education materials to support sibling coping at end of treatment

https://together.stjude.org/en-us/emotional-support-daily-life/for-siblings/how-to-help-children-when-sibling-finishes-cancer-treatment.html

Patient education materials to support social reintegration at end of treatment

School-Age Children

https://together.stjude.org/en-us/emotional-support-daily-life/parents/supporting-your-childs-friendships-after-cancer.html

Adolescents

https://together.stjude.org/en-us/emotional-support-daily-life/coping-emotional-health/friendships-after-cancer-tips-for-teens.html

Patient education materials to promote responsive caregiving for young children

https://together.stjude.org/en-us/emotional-support-daily-life/parents/responsive-caregiving-for-your-young-child.html

Collaborative creation of a podcast to support caregiver coping

https://www.stjude.org/care-treatment/patient-families/family-caregiver/caregivers-share-podcast.html
